# Bullous pemphigoid caused by contact allergy to bone cement: A case report

**DOI:** 10.1111/cod.13757

**Published:** 2020-12-27

**Authors:** Sylvie M. Franken, Thomas Rustemeyer

**Affiliations:** ^1^ Department of Dermatology Amsterdam UMC Amsterdam The Netherlands

**Keywords:** acrylate, allergy, bullous pemphigoid, case report

Localized bullous pemphigoid (BP) has been reported in up to 30% of patients with BP induced by trauma.^1^ Although there have been hypotheses about inducing the disease by traumatic triggers, the exact mechanisms remain to be elucidated. In this case report we present a patient with a possible contact allergic trigger.

## CASE REPORT

A 69‐year‐old man was seen at our Dermatology Department with complaints of blistering disease on his right leg. The complaints had started 2 to 3 weeks after knee replacement surgery on the right knee. First, small vesicles had appeared on his knee. These, however, continued to multiply and became larger in the following weeks. The blisters progressed and after few weeks resulted in large erosions that were resistant to treatment with local corticosteroids.

Skin biopsies, direct (DIF) and indirect immunofluorescence (IIF), and salt‐split skin results were all consistent with BP. Except for the right leg, there was no other skin or mucous membrane involvement.

Six months after the surgery, complaints gradually disappeared. No relapse appeared in the following year. The patient had no change in systemic medication in this period as a possible explanation of this cure. Because of the atypical course of his disease and at his specific insistence, he was referred for patch tests.

Patch tests were performed on the upper back with the European baseline series, a local extension of the baseline series, an acrylate series, and a metal series. Allergens were tested using van der Bend test chambers (Brielle, the Netherlands) applied on the upper back and covered with Fixomull stretch (BSN Medical, Hamburg, Germany). Readings were performed on day (D) 2, D4, and D7 according ESCD criteria.^2^ Tests were positive for 2‐hydroxyethyl methacrylate 2.0% pet. (D2: ?+, D4: +, D7: +), methyl methacrylate 2.0% pet. (D2: ?+, D4: +, D7: +), ethylene glycol dimethacrylate 2.0% pet. (D2: ‐, D4: +, D7: +), triethylene glycol dimethacrylate 2.0% pet. (D2: ?+, D4: +, D7: ?+), and tetrahydrofurfuryl methacrylate 2.0% pet. (D2: ?+, D4: ?+, D7: +).

Contact sensitization to several methacrylates was regarded as being of probable clinical relevance for the complaints of the patient. Prior to the knee replacement procedure, the patient had no contact with methacrylates as far as could be determined that could have caused sensitization.

## DISCUSSION

The patient presented in this case report developed complaints within weeks of articular replacement surgery. The complaints were localized at the surgery area and were suggestive of BP: clinical presentation, histopathology, DIF, and IIF confirmed this diagnosis. The complaints spontaneously disappeared without any systemic intervention approximately 6 months after developing, which is unusual for this autoimmune disease. Allergologic work‐up showed contact allergy to acrylates used in the bone cement during surgery.

Contact allergy to metals used in implants usually results in prolonged complaints^3^; problems due to acrylate allergies however are probably shorter lived. Under ideal conditions, acrylates polymerize quickly. However, a certain excess of unpolymerized monomeric methacrylates persists and for the next weeks to months after application gradually diffuses out of the bone cement and comes into the local environment.^4^ This is a self‐limiting process and consistent with our patient's clinical presentation and self‐limiting complaints.

The exact pathogenesis of BP remains unknown.^5^, ^6^ The disease is caused by immunoglobulin G and immunoglobulin E autoantibodies to BP180 and BP230, two components of the hemidesmosomes. Although there seems to be a human leukocyte antigen predisposition, there is also a role of the adaptive and innate immune system in the actual development of the blisters.^5^, ^7^, ^8^ It has been speculated that inflammation at the dermal–epidermal junction might trigger the generation of autoantibodies, resulting in the occurrence of BP.

Dǎnescu et al^1^ presented an overview of post‐traumatic BP in the literature where 31% of the cases showed a localized form of the disease. In light of our case, with a positive DIF and IIF, allergologic work up of patients with similar cases might be relevant, especially if occurring soon after surgical intervention or having a localized disease activity. One could hypothesize that this bullous disease is a presentation of a contact allergic reaction or triggered by a contact allergy as demonstrated in this case.

## CONFLICTS OF INTEREST

None.

## AUTHOR CONTRIBUTIONS

**Sylvie Franken:** Investigation; writing‐original draft. **Thomas Rustemeyer:** Investigation; writing‐review and editing.
